# Editorial: “Evolutionary Genetics of Insect Innate Immunity”

**DOI:** 10.3390/genes12050725

**Published:** 2021-05-13

**Authors:** Ioannis Eleftherianos

**Affiliations:** Infection and Innate Immunity Lab, Department of Biological Sciences, Institute for Biomedical Sciences, The George Washington University, Washington, DC 20052, USA; ioannise@gwu.edu; Tel.: +1-202-994-2367

The insect innate immune system is under strong selection pressure to evolve resistance to pathogenic infections. Previous research efforts in insect models have contributed toward the discovery of evolutionarily conserved signaling pathways and the mechanisms they regulate to trigger robust innate immune responses against pathogenic assaults. As these exciting endeavors have continued to grow in number in recent years, here we present a series of research papers and review articles that report on the progress in this research field and new advances that expand its significance.

Insects are continuously threatened by microbial enemies and interact with their microbiota. These dynamic processes shape their immune response based on the habitat they occupy. The work by Keehnen et al. reports results on how the basic immune mechanism of phagocytosis may differ genetically and phenotypically between two *Pieris napi* allopatric populations [1]. The authors describe that indeed, the types of hemocytes and their phagocytic capacity were distinct in the two butterfly populations. Further analysis of the genes that might account for the observed variability detected genomic loci that participate in glutamine metabolism. These findings indicate that changes in the genetic makeup of genes regulating immune cell differentiation could potentially be responsible for critical modifications in innate immune function in insects.

To tackle efficiently mosquito-borne diseases, it is crucial to elucidate the role of genes in the mosquito vector that control innate immune activities as well as metabolic processes that regulate general homeostasis. Here, Oringanje et al. examine the role of the AMP-activated protein kinase (AMPK) using transgenic mosquitoes and infection with *Plasmodium falciparum* [2]. The authors find that overexpression in the *Anopheles stephensi* midgut of a short version of an AMPK alpha-subunit using a carboxypeptidase promoter affects nutrient storage and metabolism after blood feeding, and decreases the levels of parasite infection. These effects are accompanied by lower production of eggs, indicating that the activity of AMPK specifically in the midgut can act as a modulator of multiple physiological functions in the mosquito vector.

Abscisic acid (ABA) is a plant hormone and conserved signaling molecule that is able to elevate the resistance of *A. stephensi* adult female mosquitoes against *Plasmodium falciparum* infection when is provided in a bloodmeal. The current work by Taylor et al. is a continuation of the previous research and attempts to confirm the effects of bloodmeal delivered ABA on *P. yoelli* in *A. stephensi* [3]. The results confirm that female adult mosquitoes obtained from ABA-treated larvae exhibit increased resistance to the parasite. This phenotype is associated with changes in the expression of insulin-like peptide and nitric oxide synthase, suggesting the transstadial impact of ABA on increasing the resistance of *A. stephensi* resistance to *Plasmodium* parasites.

Host–parasite interactions in nature can be affected when organismal behavior is modified by the presence or activity of other organisms. The molecular basis of this process involves the expression of genes coding for proteins that influence the behavior of the host or the pathogen. Here, Mangold and Hughes review specific situations where the behavioral phenotype of various insect hosts is manipulated by different microbes and parasites; they analyze the mechanisms responsible for these effects, and explain how they impact distinct aspects of the innate immune response [4]. The authors connect this information to neuroinflammation and its relationship to innate immune signaling and neural mechanisms. They further provide clues for the significance of these investigations in basic and applied research and present possibilities for expanding this type of study in order to better understand the full extent of host–microbe dynamics.

Insect immune signaling can be regulated by signal molecules, such as eicosanoids, which are critical in the integration and coordination of cellular and organismal functions. Here, Kim and Stanley [5] lay out their work on the three major groups of eicosanoids that include prostaglandins, epoxyeicosatrienoic acids and lipoxygenase products, focusing exclusively on eicosanoid biological activity in insect innate immune processes. The current review first introduces the eicosanoid biosynthesis machinery and the production of prostaglandins specifically in insects. It then provides a detailed account on recent developments in the involvement of eicosanoids in insect humoral and cellular immune activities, including gut immune responses, prophenoloxidase release and mosquito anti-*Plasmodium* reactions. Dissecting the role of eicosanoids in insect immunity allows us to explore the genetic and evolutionary origins of fundamental physiological mechanisms with immune properties in insects.

Insects form excellent models to resolve evolutionary aspects of the host anti-nematode immune response. As insects occupy different kinds of ecological niches, they are exposed to various parasitic nematodes including entomopathogenic nematodes, and insect vectors transmit filarial nematodes to new hosts. To identify the number and nature of those immune-related genes in insects that participate in the interaction with nematodes during the different stages of infection, recent research has implemented the use of state-of-the-art transcriptomic approaches. Eleftherianos and Heryanto have outlined recent progress in insect host transcriptomics as a tool to determine mechanistic immune signatures of parasitic nematode infection [6]. The presented information includes differentially regulated genes of the insect anti-nematode immunity through RNA-Seq and microarray analysis. This knowledge is critical for assisting us to clarify the genetic and evolutionary basis of insect as well as mammalian anti-nematode immunity in order to design strategies for eliminating destructive insect pests in the field.

In summary, the current Special Issue “Evolutionary Genetics of Insect Innate Immunity” reports novel information that expands from the population genomics of cellular immunity in butterflies to evolutionarily conserved genetic mechanisms of malaria resistance in mosquitoes, and from dissecting the connection between neural-mediated behavioral manipulation and the role of eicosanoid signaling in insect immunity to understanding the insect transcriptomic response to parasitic nematode infection. This knowledge sets the stage for exciting future investigations that will further extend the frontiers of insect immunology.
